# Interactions between *Schistosoma haematobium* group species and their *Bulinus* spp. intermediate hosts along the Niger River Valley

**DOI:** 10.1186/s13071-020-04136-9

**Published:** 2020-05-24

**Authors:** Tom Pennance, Fiona Allan, Aidan Emery, Muriel Rabone, Jo Cable, Amadou Djirmay Garba, Amina Amadou Hamidou, Joanne P. Webster, David Rollinson, Bonnie L. Webster

**Affiliations:** 1 Department of Life Sciences, Natural History Museum, Cromwell Road, South Kensington, London, SW7 5BD UK; 2 School of Biosciences, Cardiff University, Cardiff, CF10 3AX UK; 3 London Centre for Neglected Tropical Disease Research, Imperial College London, School of Public Health, Norfolk Pl, Paddington, London, W2 1PG UK; 4 Réseau International Schistosomoses, Environnement, Aménagement et Lutte (RISEAL-Niger), 333, Avenue des Zarmakoye, B.P. 13724 Niamey, Niger; 5 World Health Organization, Geneva, Switzerland; 6 Department of Pathology and Pathogen Biology, Centre for Emerging, Endemic and Exotic Diseases (CEEED), Royal Veterinary College, University of London, Hertfordshire, AL9 7TA UK

**Keywords:** *Schistosoma haematobium*, *Schistosoma bovis*, Hybrids, Urogenital schistosomiasis, *Bulinus globosus*, *Bulinus truncatus*, *Bulinus forskalii*, Cercariae, Niger, Molecular identification

## Abstract

**Background:**

Urogenital schistosomiasis, caused by infection with *Schistosoma haematobium,* is endemic in Niger but complicated by the presence of *Schistosoma bovis*, *Schistosoma curassoni* and *S. haematobium* group hybrids along with various *Bulinus* snail intermediate host species. Establishing the schistosomes and snails involved in transmission aids disease surveillance whilst providing insights into snail-schistosome interactions/compatibilities and biology.

**Methods:**

Infected *Bulinus* spp. were collected from 16 villages north and south of the Niamey region, Niger, between 2011 and 2015. From each *Bulinus* spp., 20–52 cercariae shed were analysed using microsatellite markers and a subset identified using the mitochondrial (mt) *cox*1 and nuclear ITS1 + 2 and *18S* DNA regions. Infected *Bulinus* spp. were identified using both morphological and molecular analysis (partial mt *cox*1 region).

**Results:**

A total of 87 infected *Bulinus* from 24 sites were found, 29 were molecularly confirmed as *B. truncatus*, three as *B. forskalii* and four as *B. globosus.* The remaining samples were morphologically identified as *B. truncatus* (*n* = 49) and *B. forskalii* (*n *= 2). The microsatellite analysis of 1124 cercariae revealed 186 cercarial multilocus genotypes (MLGs). Identical cercarial genotypes were frequently (60%) identified from the same snail (clonal populations from a single miracidia); however, several (40%) of the snails had cercariae of different genotypes (2–10 MLG’s) indicating multiple miracidial infections. Fifty-seven of the *B. truncatus* and all of the *B. forskalii* and *B. globosus* were shedding the Bovid schistosome *S. bovis.* The other *B. truncatus* were shedding the human schistosomes, *S. haematobium* (*n* = 6) and the *S. haematobium* group hybrids (*n* = 13). Two *B. truncatus* had co-infections with *S. haematobium* and *S. haematobium* group hybrids whilst no co-infections with *S. bovis* were observed.

**Conclusions:**

This study has advanced our understanding of human and bovid schistosomiasis transmission in the Niger River Valley region. Human *Schistosoma* species/forms (*S. haematobium* and *S. haematobium* hybrids) were found transmitted only in five villages whereas those causing veterinary schistosomiasis (*S. bovis*), were found in most villages. *Bulinus truncatus* was most abundant, transmitting all *Schistosoma* species, while the less abundant *B. forskalii* and *B. globosus,* only transmitted *S. bovis.* Our data suggest that species-specific biological traits may exist in relation to co-infections, snail-schistosome compatibility and intramolluscan schistosome development. 
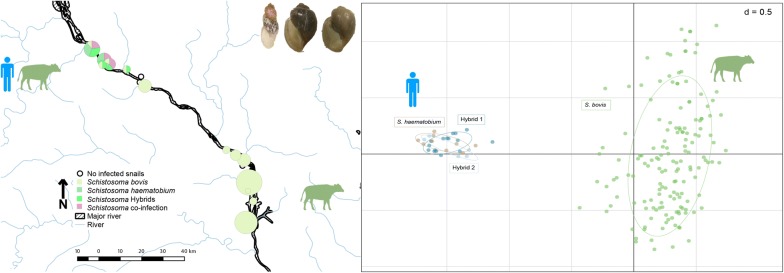

## Background

Schistosomiasis is a snail-borne neglected tropical disease (NTD) associated with poverty, poor sanitation and lack of safe water [1, 2]. An estimated 180–200 million people are infected, primarily from tropical low and middle-income countries [3]. Schistosomes are diverse, with 25 species currently recognised within the genus *Schistosoma*, and exhibit preferences for both their intermediate snail and mammalian hosts, both of which governs their distribution. In sub-Saharan Africa, there are four species that cause human schistosomiasis, whilst there are many more that infect livestock and/or wildlife [4].

Schistosomes can be identified by species-specific phenotypic characteristics, particularly associated with the adult worms and their eggs. Additionally, epidemiological and ecological characteristics, such as geographical region and intermediate snail and definitive mammalian host associations, are used as proxies for species identification at a given focus [5]. Supported by new field collection and sample preservation methods [6, 7], molecular data from schistosome collections have revealed new species distributions [8], interspecies hybridisation [9–12], and unexpected host associations [13], all of which highlight the need to incorporate molecular analyses into disease surveillance. Molecular data are particularly pertinent for free-living schistosome cercarial larvae, which have limited species-specific morphologies [14] and also for schistosomes that overlap in their snail intermediate host use.

Aside from schistosomes, snail host identification can pose complications as it often relies on species-specific morphological characteristics, including shell characteristics, morphology of reproductive organs and chromosome counts [15]. However, morphologically similar snails can be very difficult to identify, with phenotypic plasticity, including overlap in shell morphology potentially confounding accurate identifications [16]. Molecular analyses provide greater accuracy and also enable interrelationship, phylogenetic and population diversity analyses [17]. Moreover, precise snail identifications are needed to determine the true hosts involved in *Schistosoma* spp. transmission. This is vital for mapping and monitoring disease transmission and determining infection risk to human and livestock populations surrounding snail habitats [18], which in addition will also aid in determining suitable localities for targeted snail control, both of these points being listed by the World Health Organisation as critical actions necessary for reaching the goal of eliminating schistosomiasis as a public health problem by 2030 [19].

Of the estimated 3–4 million people at risk of human schistosomiasis in Niger, the majority of the disease is urogenital, caused by *Schistosoma haematobium* (including *S. haematobium* group hybrids), with transmission relying on freshwater *Bulinus* spp. snails [20, 21]. There is also a relatively unknown burden of veterinary schistosomiasis caused by the schistosomes *Schistosoma bovis* and *Schistosoma curassoni*, and also *S. bovis*-*curassoni* hybrids [22–24], all of which are transmitted by *Bulinus* spp. [22, 23, 25]. Additionally, the occurrence of *S. haematobium* group hybrids, involving a mixture of human and veterinary schistosome species (*S. haematobium*-*S. bovis*, *S. haematobium*-*S. curassoni*, *S. haematobium*-*S. bovis*-*S. curassoni*) have been reported from humans in Niger [10, 24, 26], and are reported in other African countries [9, 11, 27–31]. This complicates transmission and epidemiology, while raising many questions regarding the general biology of both snail and mammalian host specificities of these closely related schistosome species.

Our current understanding of *S. haematobium* and *S. bovis* interactions with the intermediate hosts in Niger is based on morphological identifications (cercariae and their snail host), cercarial emergence patterns, and experimental infections. Regarding the intermediate hosts of human schistosomiasis, *Bulinus senegalensis*, *Bulinus forskalii* and *Bulinus truncatus* have previously been reported to transmit *S. haematobium* [32–36] while *Bulinus umbilicatus* and *B. senegalensis* have been reported as shedding the veterinary schistosomes, *S. curassoni* and *S. bovis*, respectively [25, 34]. However, molecular data are needed to support these predominantly morphological observations for both snail and schistosome, providing a more accurate assessment of snail-schistosome relations and epidemiology [10, 32].

Using a combination of methods, this study investigates the snail-schistosome relationships of *S. haematobium* group species and their *Bulinus* snail hosts along the Niger River Valley to: (i) identify the *Bulinus* species involved in schistosomiasis transmission in the Niamey region of Niger; (ii) identify the schistosome species being transmitted by specific *Bulinus* spp. in these areas; and (iii) investigate schistosome single- and multiple species/genotypes co-infections within individual snails.

## Methods

### Study area and sample collection

This study was part of the urogenital schistosomiasis control project in Niger, incorporated into the wider Schistosomiasis Consortium for Operational Research and Evaluation (SCORE) programme [37]. Malacological surveys coupled with snail schistosome infection screening were conducted at potential urogenital schistosomiasis transmission sites, in 16 villages located approximately 60 km upstream (North) and downstream (South) of Niamey, along the Niger River basin.

In total, 68 potential transmission/water contact sites were surveyed monthly from July 2011 to January 2016, a subset of those reported in Rabone et al. [38]. At each site, two collectors searched and collected snails, morphologically identified as *Bulinus* spp. [15] using scoops and by picking them directly from freshwater vegetation for 15–30 min. Individual snails were then checked for patent schistosome infections by cercarial shedding. Furcocercous cercariae were morphologically identified using descriptions of *Schistosoma* spp. [14], and collected and preserved for molecular characterisation on Whatman-Indicating FTA Classic Cards (GE Healthcare Life Sciences, Buckinghamshire, UK) by pipetting individual cercariae onto the card in 2–3 µl [30], and the corresponding infected snails were preserved in 100% ethanol for future molecular identification.

All information on collection dates and site localities containing infected snails can be found in Additional file 1: Table S1 and Rabone et al. [38]. Primers and PCR cycling conditions used for molecular characterisation and identification of the schistosome cercariae and the *Bulinus* snail hosts, are described in Additional file 2: Table S2.

### Molecular identification and mitochondrial *cox*1 analyses of the schistosome-infected *Bulinus* spp.

The soft tissue of the individual snails was removed from the shell, and DNA extracted using the Qiagen DNeasy Blood and Tissue Kit (Qiagen, Manchester, UK) as described in Pennance et al. [8]. Each snail was identified by analysis of a partial mitochondrial *cox*1 DNA region. Due to the genetic diversity within and between *Bulinus* spp., different primer combinations and PCR cycling conditions were used as described in Additional file 2: Table S2 [17, 39]. The primer Primer Bglob_CoxF (Additional file 2: Table S2) was designed manually for use in this study using the reference *B. globosus* (Niger strain) *cox*1 data (GenBank: AM286294, Kane et al. [17]). For *Bulinus* spp., PCRs were performed using PuReTaq Ready-To-Go PCR beads (GE Healthcare Life Sciences, Buckinghamshire, UK) in 25 µl reactions composed of 1 µl of the snail DNA and 1 µl of each primer at a concentration of 10 µmol. Amplicons (4 μl) were visualised by gel electrophoresis on a 2% GelRed agarose gel and positive reactions were purified using the QiaQuick PCR Purification Kit (Qiagen).

Amplicons were Sanger sequenced in both directions using a dilution of the original PCR primers on an Applied Biosystems 3730xl DNA analyser and the edited *cox*1 data from each sample was analysed using the NCBI BLASTn search to confirm species identity. Additionally, each *cox*1 nucleotide sequence was translated (using the invertebrate mitochondrial genetic code) and the resulting amino acid sequence analysed using the NCBI BLASTx search to further confirm species identity and to check for the occurrence of non-coding data which indicates the presence of mitochondrial pseudogenes. This was performed due to nucleotide deletions observed in the *cox*1 regions from some of the samples.

The *cox*1 consensus sequence data from each snail were aligned in Sequencher v5.4.6 (GeneCodes Corp., Michigan, USA) and then collapsed together to identify individuals with identical sequences (haplotypes). The number of unique *cox*1 haplotypes was recorded for each *Bulinus* species. A haplotype network was generated using PopART [40] to investigate haplotype relationships and diversity.

Haplotype sequences were imported into Geneious v11.1.4 [41] for phylogenetic analysis together with reference data available from GenBank; Nigerien *Bulinus* spp. accession numbers: AM286308 (*B. forskalii*), AM286294 (*B. globosus*), AM286316 (*B. truncatus*), AM286317 (*B. truncatus*) (see [17]). *Biomphalaria glabrata* (AY380531; see [42]) was included as the outgroup. Haplotype alignments were performed using the MAFFT v7.388 [43, 44], MUSCLE v3.8.425 [45] and Clustal W v2.1 [46] tools in Geneious v11.1.4 [41]. Alignments were executed in PAUP* [47] and then an appropriate evolutionary nucleotide substitution model was selected in MrModelTest v2.4 [48] using the Akaike Information Criterion. Bayesian inference analysis and phylogenetic tree construction was performed using MrBayes v3.2.7a [49] under the GTR + G model for 5 million generations. The ‘burn-in’ was defined as the point at which the average standard deviation of split frequencies (ASDOSF) reported from MrBayes output was at least < 0.01 over 5 million generations, although a ‘burn-in’ of 3.5 million generations was used for consistency. Clades were considered to have high nodal support if Bayesian inference posterior probability was ≥ 0.95, therefore tree nodes with < 0.95 were collapsed in SumTrees v4.4.0 [50] to give the final tree topology.

### Genetic profiling and molecular species identification of the schistosome cercariae

DNA was prepared from individual cercariae on FTA cards as previously described [7]. A minimum of 20 (except for one snail, where only 4 cercariae were recovered) and a maximum of 52 cercariae were analysed from each shedding snail (Additional file 1: Table S1). Individual cercariae were genotyped using the previously developed [7] Panel 1 microsatellite loci (Additional file 3: Table S3). The microsatellite reactions were performed using the Type-it**®** Microsatellite PCR Kit (Qiagen) in 12.5 μl reactions containing 2 µl of alkaline eluted schistosome DNA, 0.2 μM of each primer and 1.25 μl of the Type-it**®** Microsatellite PCR Kit Q-Solution. The primers and PCR cycle are the same as those reported in Webster et al. [7]. Amplicons (4 μl) were visualised by gel electrophoresis on a 3% GelRed agarose gel. Positive reactions were diluted 1 in 10 before being denatured and injected at an optimal speed of 12 s into the Applied Biosystems 3130xl DNA analyser for analysis.

Allele calls were checked and edited in Geneious v11.1.4 [41] using the microsatellite plugin (Biomatters Ltd. v1.4.6). Out of the nine microsatellite loci, six provided good quality data for all cercariae analysed. The three loci (C102, C111 and Sh7) not included in the analysis proved species-specific to *S. haematobium* (Additional file 1: Table S1). Matching cercarial microsatellite profiles from each snail (indicating clonal cercariae) were then grouped together into individual MLGs for each infected snail. We recorded the number of different cercarial MLG’s originating from each snail and the number of cercariae within each MLG. To check for potential sampling bias in relation to the numbers of cercarial MLG’s found per snail, a Pearson’s correlation test was performed in R version 3.5.2 [51] comparing the number of cercarial genotypes observed per infected *Bulinus* spp. with the number of cercariae analysed.

### Schistosome cercariae species and hybrid identification

Both mitochondrial and nuclear DNA regions were analysed to determine the schistosome species or hybrid status of each MLG [9, 27]. The diagnostic *cox*1 PCR, that distinguishes between *S. bovis* and *S. haematobium* based on the size of the *cox*1 amplicon [52], was used to screen all the selected cercariae (Additional file 1: Tables S1, Additional file 2: Table S2). A subset of the amplicons were sequenced to confirm the mitochondrial genotypes and to enable preliminary haplotype analyses.

The nuclear ITS1 + 2 rDNA region (including the *5.8S* gene region) was also amplified (~ 915 bp) (Additional file 2: Table S2) and sequenced to identify the species-specific SNP’s (Additional file 4: Table S4). Additionally, a fragment of the 5’-end of a second rDNA region (*18S*) [53] containing additional species-specific SNPs (Additional file 4: Table S4) was amplified (289 bp) (Additional file 2: Table S2) and sequenced for a subset of the cercariae.

For *Schistosoma* spp., PCRs of *cox*1, ITS and *18S* were performed using PuReTaq Ready-To-Go PCR beads (GE Healthcare Life Sciences, Buckinghamshire, UK) in 25 µl reactions composed of 3 µl of alkaline eluted schistosome DNA and 1 µl of each primer at a concentration of 10 µmol. Amplicons (4 μl) were visualised by gel electrophoresis on a 2% GelRed agarose gel and positive reactions were purified using the QiaQuick PCR Purification Kit (Qiagen). Amplicons were Sanger sequenced in both directions using a dilution of the original PCR primers on an Applied Biosystems 3730xl DNA analyser. Sequence identity was confirmed using NCBI BLASTn and/or by comparison to reference data [51]. Following successful sequencing and analysis, both mitochondrial (*cox*1) and nuclear (ITS + *18S*) profiles were assigned to each cercarial MLG to determine species or hybrid status.

### *Schistosome cercariae* microsatellite population structure analysis

To gain insights into the population structure of the cercariae, the microsatellite data were subjected to principal components analysis (PCA) using the *adegenet* v2.1.1 package [54] in R version 3.5.2 [51]. PCA was performed on two datasets with all loci including the missing data (from the 3 *S. haematobium-*specific loci C102, C111, Sh7) and excluding the missing data. Each cercarial MLG analysed was grouped into the *Schistosoma* spp. identified: *Schistosoma haematobium*, *S. bovis* or *S. haematobium* group hybrids. Analyses were run both including and excluding missing microsatellite loci data which occurred due to the specificity of the microsatellite primers to *S. haematobium*.

### Distribution of infected *Bulinus* and *Schistosoma* species

*Bulinus* spp. and *Schistosoma* spp. distribution data were visualised using QGIS v3.0.1 Girona (http://qgis.osgeo.org). Collection sites were grouped by village and points scaled to the number of infected *Bulinus* spp. Digital shape files for Niger administrative regions and inland water areas were obtained from DIVA-GIS (https://www.diva-gis.org).

## Results

### Distribution and abundance of the *Schistosoma*-infected *Bulinus* species

**Fig. 1:**
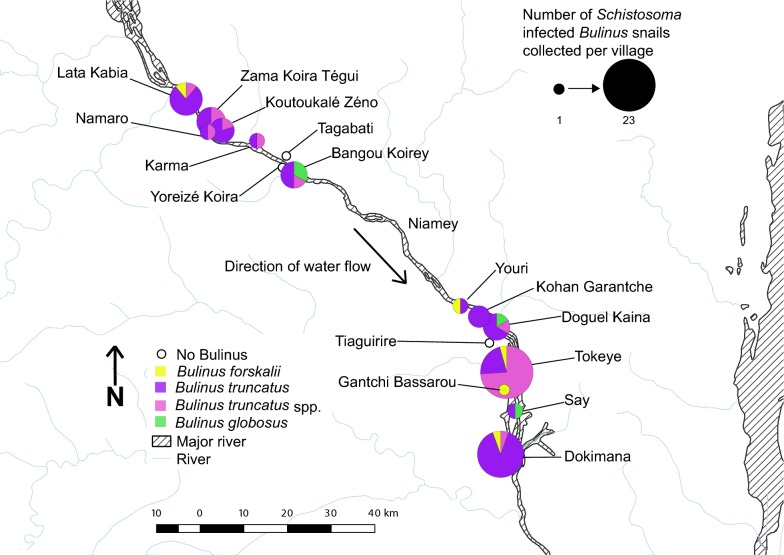
Distribution of infected *Bulinus* spp. snails in Niamey region of Niger. Circle size is proportional to the number of infected *Bulinus* spp. per village

Of the 15,288 *Bulinus* spp. snails collected from the study sites in Niger, 137 had patent schistosome infections (0.90%). These were collected at multiple time points between 2011 and 2015 from 26 of the 68 sites surveyed from villages both in the North and South of Niamey (Fig. 1). Infected *Bulinus* spp. were found in several different habitat types surveyed: rivers, ponds, streams, rice paddies, secondary irrigation canals, tertiary irrigation canals, branching streams and spillways. *Schistosoma* cercariae and snail molecular data were obtained from 87 of the 137 infected *Bulinus* spp. collected from 24 of the 68 sites (50 snails could not be included in the study due to specimen degradation, Table 1).

**Table 1:** *Schistosoma* spp. infections identified from *Bulinus* snails identified using morphology and molecular techniques from Niger

Village	No. of infected *Bulinus* spp.	*Bulinus* with each *Schistosoma* infection				*Bulinus* species identified using molecular and morphological methods			
		*S. bovis*	*S. haematobium*	*S. haematobium* group hybrids	Co-infection^a^	*B. forskalii* (mol/mor)	*B. globosus* (mol/mor)	*B. truncatus* (mol/mor)	*B. truncatus* spp.^b^
Zama Koira Tégui	7	0	2	3	2	0/0	0/0	2/1	4
Yoreizé Koira	0	0	0	0	0	0/0	0/0	0/0	0
Tagabati	0	0	0	0	0	0/0	0/0	0/0	0
Bangou Koirey	6	6	0	0	0	0/0	2/0	3/0	1
Namaro	2	1	0	1	0	0/0	0/0	1/0	1
Lata Kabia	9	1	3	3	2	1/0	0/0	3/4	1
Koutoukalé Zéno	5	1	1	1	2	0/0	0/0	0/4	1
Karma	2	1	0	1	0	0/0	0/0	0/1	1
Youri	2	2	0	0	0	0/1	0/0	0/1	0
Tokèye	23	23	0	0	0	0/1	0/0	1/4	17
Gantchi Bassarou	1	1	0	0	0	1/0	0/0	0/0	0
Dokimana	18	18	0	0	0	1/0	0/0	12/4	1
Say	2	2	0	0	0	0/0	1/0	0/1	0
Kohan Garantche	4	4	0	0	0	0/0	0/0	4/0	0
Doguel Kaina	6	6	0	0	0	0/0	1/0	3/1	1
Tiaguirire	0	0	0	0	0	0/0	0/0	0/0	0
Total (North)	31	10	6	9	6	1/0	2/0	9/10	9
Total (South)	56	56	0	0	0	2/2	2/0	20/11	19
Total (Overall)	87	66	6	9	6	3/2	4/0	29/21	28

mol, molecularly confirmed; mor, morphologically confirmed

^a^Co-infected snails shedding more than one schistosome species/hybrid type

^b^*Bulinus truncatus* spp. based on morphology and preferential amplification of non-coding mitochondrial genes

### Molecular identification and *cox*1 analyses of the schistosome-infected *Bulinus* species

Of the 87 infected snails included in the analyses, 82 were morphologically identified at the time of collection as *B. truncatus* and five as being from the *B. forskalii* group (Table 1). Molecular analysis of 61 of the morphologically identified *B. truncatus* confirmed that 57 were *B. truncatus* but four were *B. globosus*. Identification of three of the five *B. forskalii* was also confirmed by molecular data. For the remaining 23 infected *Bulinus* spp., poor DNA quality precluded molecular analysis and therefore identifications were made solely by morphology (Table 1): *B. forskalii* (*n *= 2); *B. truncatus* (*n *=21). The geographical distributions of the different snail species are shown in Fig. 1.

Eight different *cox*1 haplotypes were identified from 29 of the 57 infected *B. truncatus* (Table 2). For the remaining 28 *B. truncatus* specimens, only non-coding mitochondrial *cox*1 pseudogenes were generated. These data could not be translated (contained many stop codons) and contained deletions (10–18 bp). Attempts to obtain the functional partial *cox*1 DNA region, using alternative primers and combinations, either failed to amplify the target region, or produced mixed sequence profiles. The resulting data, however, were sufficient to confirm the snail species as within the *B. truncatus* species complex (see Table 1). The three *B. forskalii* fell into two haplotypes and the four *B. globosus* fell into three different haplotypes (Table 2). The *Bulinus cox*1 haplotype network revealed clear species division and a diverse population with no structuring or dominant haplotypes (Additional file 5: Figure S1). Phylogenetic tree topology (Additional file 6: Figure S2) showed the three expected monophyletic groups: (i) *B. forskalii*; (ii) *B. globosus*; and (iii) *B. truncatus.* There was no support for significant intraspecies variation between the haplotypes in each clade, further confirming species assignment.

**Table 2:** *Bulinus* species haplotypes identified and the numbers of snails representing each with GenBank accession numbers

Species	*Bulinus* spp. haplotype	No. of samples	Sequence length (bp)	GenBank ID
*Bulinus truncatus*	*B. truncatus* 1	3	653	MT272327
	*B. truncatus* 2	10	652	MT272328
	*B. truncatus* 3	7	653	MT272329
	*B. truncatus* 4	4	636	MT272330
	*B. truncatus* 5	2	653	MT272331
	*B. truncatus* 6	1	653	MT272332
	*B. truncatus* 7	1	397	MT272333
	*B. truncatus* 8	1	636	MT272334
	*B. truncatus* 9 (nc)	6	na	na
	*B. truncatus* 10 (nc)	16	na	na
	*B. truncatus* 11 (nc)	3	na	na
	*B. truncatus* 12 (nc)	3	na	na
*Bulinus forskalii*	*B. forskalii* 1	2	651	MT272322
	*B. forskalii* 2	1	651	MT272323
*Bulinus globosus*	*B. globosus* 1	2	600	MT272325
	*B. globosus* 2	1	589	MT272326
	*B. globosus* 3	1	414	MT272324

nc, probable translocated pseudogenes; na, non-protein-coding DNA not included in analysis or submitted to GenBank

### Schistosome cercariae microsatellite analysis

**Fig. 2:**
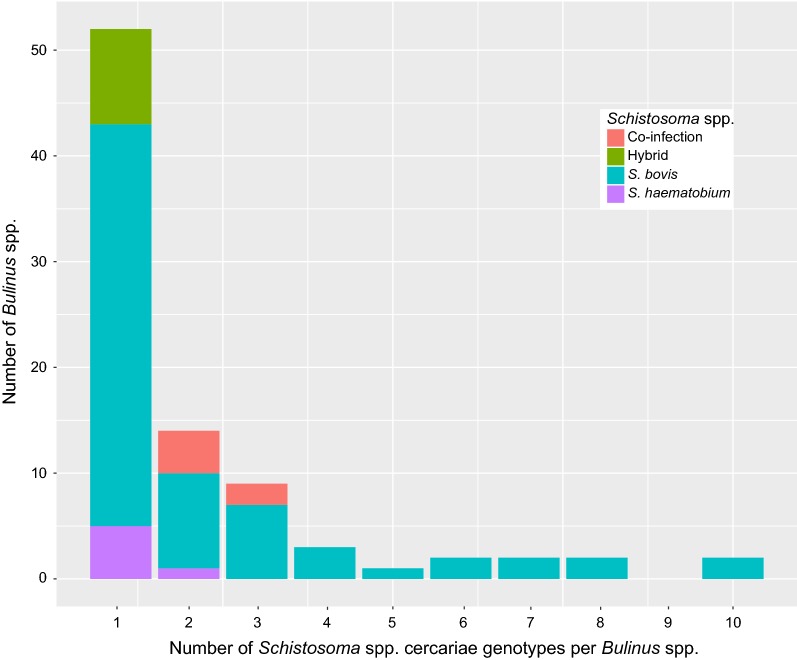
Number of unique *Schistosoma* spp. multilocus microsatellite genotypes per *Bulinus* spp

In total, 1124 cercariae were characterised from the 87 infected *Bulinus* spp. (Additional file 1: Table S1), identifying 186 unique cercarial multilocus genotypes (MLG’s). Cercarial genotypes differed between snails, with identical genotypes frequently identified from individual cercariae shed from the same snail, due to the clonal replication of the schistosome larval stages (Additional file 1: Table S1). However, in several cases cercariae originating from the same snail had different genotypes, indicating that they had co-infections of the same species or different species. The number of different cercarial MLGs per individual snail ranged from 1 to 10 (Fig. 2), with 52 (60%) of the infected *Bulinus* shedding cercariae with a single MLG and 35 (40%) shedding cercariae with > 1 MLG. Sample size analyses showed that the number of cercarial genotypes observed per infected *Bulinus* spp. was not dependent on the number of cercariae analysed (see Additional file 7: Figure S3, Pearson’s correlation coefficient, *r* = 0.04).

### Schistosome cercariae species/hybrid identification

Of the infected snails, 66 (76%) were shedding the livestock schistosome *S. bovis* only, six (7%) were shedding the human schistosome *S. haematobium* and 9 (10%) the *S. haematobium* group hybrids. The remaining six snails (7%) harboured co-infections with different *S. haematobium* group hybrids and/or *S. haematobium* (Table 3).

**Table 3:** *Schistosoma haematobium* group infections observed in *Bulinus* spp. from Niger villages

*Bulinus* spp.	Snail-*Schistosoma* spp. infection profile	No. of snails	Suspected definitive host
*B. truncatus*	*S. bovis*	57	Bovidae
	*S. haematobium*	6	Human
	Hybrid 1 (*S.h*-*b*)	9	Human
	Co-infection: *S. haematobium *+Hybrid 1 (*S.h*-*b*)	1	Human
	Co-infection: *S. haematobium *+Hybrid 2 (*S.h*-*b*-*c*)	1	Human
	Co-infection: Hybrid 1 (*S.h*-*b*)+Hybrid 2 (*S.h*-*b*-*c*)	4	Human
*B. forskalii*	*S. bovis*	5	Bovidae
*B. globosus*	*S. bovis*	4	Bovidae

Of the hybrid cercariae identified, two hybrid genetic profiles were observed (Additional file 4: Table S4). The most common hybrid (*S. haematobium*-*bovis*, *S.h*-*b*), displaying a *S. bovis* mt *cox*1 and *S. haematobium* nuclear profile, was shed from 14 (16%) of the infected *Bulinus.* The second hybrid genetic profile (*S. haematobium*-*bovis*-*curassoni*, *S.h*-*b*-*c*) was more unusual, consisting of a *S. bovis* mt *cox*1 and a mixed *S. haematobium*/*S. curassoni* nuclear profile [10]. The unusual genetic profiles of these human-infecting schistosomes were further confirmed by analysis of the partial 5’-end of the *18S* nuclear ribosomal gene region, which contains further *S. bovis*/*S. curassoni* interspecies SNP’s (Additional file 4: Table S4). The following schistosome co-infections were observed in the snails: *S. haematobium* + hybrid 1 (*S.h*-*b*); *S. haematobium* + hybrid 2 (*S.h*-*b*-*c*); or hybrid 1 (*S.h*-*b*) + hybrid 2 (*S.h*-*b*-*c*). Single-species/hybrid infections of snails were observed with hybrid 1 (*S.h*-*b*), *S. haematobium* or *S. bovis.* No multiple species co-infections were found in snails infected with *S. bovis.*

*Cox*1 haplotype data were obtained from 18 *S. bovis cox*1 amplicons, of which 15 were from cercariae identified as *S. bovis* and three from hybrid cercariae (*S.h*-*b* and *S.h*-*b*-*c*). Four *S. bovis* mt *cox*1 haplotypes were identified (Sb-Hap-1, GenBank: MT272336; Sb-Hap-2, GenBank: MT272338; Sb-Hap-3, GenBank: MT272337; Sb-Hap-4, GenBank: MT272339), with all the hybrid cercariae presenting the same *cox*1 haplotype (Sb-Hap-2) whereas all the *S. bovis* cercariae presented all haplotypes (Sb-Hap-1-4). Six *S. haematobium* cercariae shed from five *B. truncatus* presented a single *cox*1 haplotype (Sh-Hap-1, GenBank: MT272340). The *S. haematobium* mt *cox*1 haplotype is a common haplotype found across Africa representing the H1 pan-African haplotype [55]. Cercariae with multiple *cox*1 haplotypes were also observed from cercariae shed from the same snail (see snail: LA79, Additional file 1: Table S1), supporting the observations of multiple mono-species infections of individual snails inferred from cercarial MLG data.

### *Schistosoma* species and hybrid associations with the different *Bulinus* species

All of the *B. forskalii* (*n* = 5), *B. globosus* (*n* = 4) and 73% of the *B. truncatus* (*n* = 57) were only shedding *S. bovis* cercariae (Table 3). Six of the *B. truncatus* (8%) were only shedding cercariae of *S. haematobium* and nine of the *B. truncatus* (12%) were only shedding cercariae of the *S. haematobium*-*bovis* hybrid 1 (*S.h*-*b*). All other *B. truncatus* (*n* = 6) had co-infections, four involving both hybrid 1 (*S.h*-*b*) + hybrid 2 (*S.h*-*b*-*c*), one involving *S. haematobium* + hybrid 1 (*S.h*-*b*) and one involving *S. haematobium* + hybrid 2 (*S.h*-*b*-*c*) (Table 3). No co-infections or hybrid infections were observed in *B. globosus* or *B. forskalii*.

### Multilocus genotypes and snail/schistosome species associations

The number of cercarial MLGs per snail showed differences by snail species, for example; two *B. truncatus* were found shedding *S. bovis* cercariae of up to ten MLGs, one *B. forskalii* was shedding *S. bovis* cercariae with up to six MLGs whereas all four *B. globosus* snails were shedding *S. bovis* cercariae of a single genotype.

The highest numbers of single-species MLG’s found per snail were associated with *S. bovis* infections, indicating potential multiple co-infections with different *S. bovis* miracidia (Fig. 2). A wide range of these single-species multiple genotype co-infections with different *S. bovis* miracidia were observed, with MLG’s per snail ranging from 1 to 10.

For the single-species infections involving *S. haematobium* or the *S. haematobium* group hybrids, only low numbers of MLG’s were identified per snail (1 or 2 MLG per snail for *S. haematobium* and 1 per snail for *S. haematobium* group hybrids). This indicates that infections only involved 1 or 2 miracidia for these species/hybrids (Fig. 2).

### Microsatellite population structure of human and bovine schistosome cercariae

**Fig. 3:**
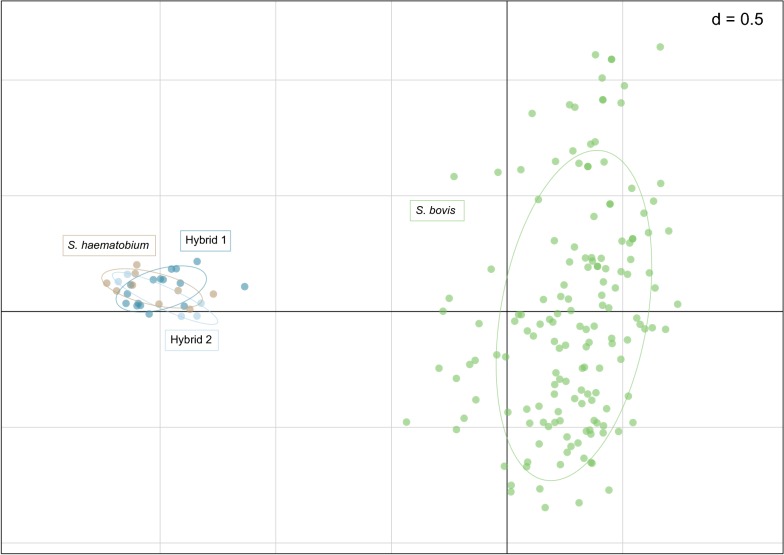
Principal components analysis biplot of the *Schistosoma* spp. cercariae allele frequencies shed from *Bulinus* spp. PCA produced using 6 microsatellite loci (excluding 3 *Schistosoma haematobium-*specific loci) of *Schistosoma* spp. cercariae shed from *Bulinus* spp. collected in the Niger River Valley (186 observations). The cumulative proportion of explained variation is 19.8% for the first two principal components (PC1: 14.1%; PC2: 5.7%). Observations are coloured by *Schistosoma* species profile (see Additional file 1: Table S1)

PCA analysis including and excluding missing data showed the same population clustering patterns. The PCA plot reveals two population clusters one that included *S. haematobium* and the *S. haematobium* group hybrids and a second that included only *S. bovis* suggesting no gene flow between the populations (Fig. 3).

### Distribution of infected *Bulinus* and the *Schistosoma* species

**Fig. 4:**
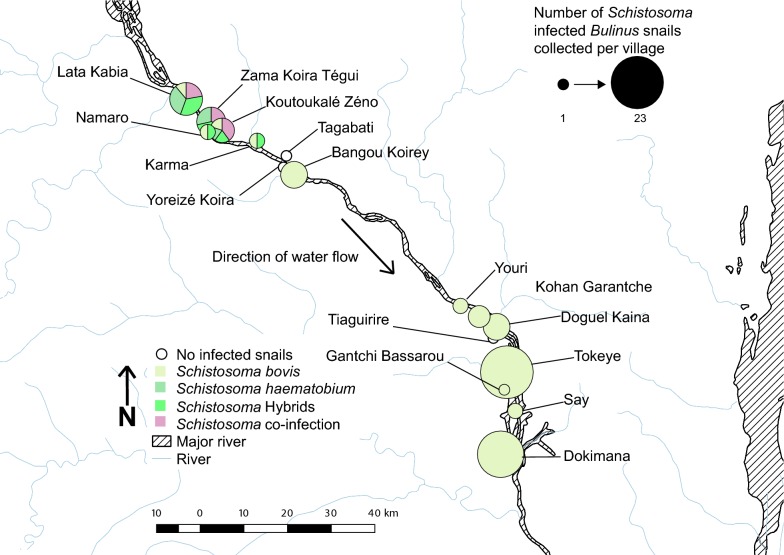
Distribution of *Schistosoma* spp. cercariae shed from *Bulinus* spp. collected in Niamey region, Niger River Valley. Circle size is proportional to the number of infected *Bulinus* spp. per village

*Schistosoma haematobium* and the *S. haematobium* group hybrids involved in human urogenital schistosomiasis infections were only detected in five villages north of Niamey (Fig. 4). *Schistosoma bovis* occurred throughout the study villages in the north and south (except Yoreizé Koira and Tagabati) but was the only schistosome species found in the southern sites, where the majority (*n* = 56) of infected snails were collected (Table 1), predominantly being *B. truncatus* collected from two villages: Tokeye (*n* = 23) and Dokimana (*n* = 18) (Fig. 2). Fewer snails were involved in the transmission of human urogenital schistosomiasis (*n* = 21) compared to those involved in the transmission of the bovid schistosomes (Table 3). The infected *B. globosus* and *B. forskalii* were present in low numbers in both the northern and southern sites, transmitting *S. bovis*.

## Discussion

Molecular identification of schistosome cercariae and their snail hosts has proved vital for identifying the species of schistosome being transmitted in the Niger River Valley, enabling the mapping of human and animal schistosomiasis transmission and risk. Particularly, the fine scale analyses were able to identify transmission sites that were specifically involved in human schistosomiasis transmission. This supports more targeted interventions towards these sites and the local communities at high risk of infection. Additionally, unravelling the relationships between the different schistosome species and their snail host species at a focal level allows us to gain a better understanding of disease transmission and snail-schistosome epidemiology. Here we have identified the transmission of *S. haematobium* and *S. bovis,* causing human and livestock schistosomiasis, respectively, in the Niger River Valley region (Fig. 4). We also detected *S. haematobium-bovis* hybrids, known human pathogens, adding to the geographical range of these hybrids now reported in several countries, Senegal, Niger, Mali, Côte d’Ivoire, Malawi [10, 27, 31, 56] and also imported into Corsica (France) [57]. Additionally, *S. haematobium*-*bovis*-*curassoni* hybrid cercariae were identified, confirming the transmission of this unusual hybrid combination previously reported from humans in Niger [10].

The microsatellite data analysis showed no gene flow between the human (*S. haematobium* and the hybrids) and cattle (*S. bovis*) schistosome populations analysed (Fig. 3). This suggests that strong reproductive barriers exist between the two populations and that the observed hybrids are not first-generation hybrids resulting from zoonotic and/or zooanthroponotic interspecies interactions. These hybrids appear to be introgressed forms, with parts of the *S. bovis* genome introgressed into *S. haematobium*, leaving two differentiated parental populations that are not panmictic or leading to hybrid speciation. This was also reported in recent studies in Senegal [58] and Niger [59], and indicates that more research is warranted to understand *S. haematobium* group species hybridisation, the effect of hybridisation on definitive host range [13] and the potential human and veterinary impacts [60].

Mitochondrial *cox*1 analysis of the *Bulinus* snail samples identified the three species, *B. truncatus*, *B. globosus* and *B. forskalii*, involved in schistosome transmission in Niger. These three *Bulinus* species show diverse intraspecies populations, with no clustering by geographical region or in relation to transmission. Additionally, non-coding mitochondrial DNA was amplified in several of our *B. truncatus* samples, the preferential sequencing of which could be attributed to unsuitable primer selection or specimen degradation. As these were non-coding, they cannot be linked to NUMTs described in other molluscs [61] and may instead be related to the high degree of polyploidy known to occur in *B. truncatus*/*tropicus* group snails [62–64] promoting mitochondrial heteroplasmy. Although mitochondrial barcoding is useful for exploring genetic diversity and relationships of *Bulinus* spp. [17, 65], the preferential sequencing of non-coding genes here highlights difficulties in using such a marker for species identification and the need for using other informative DNA regions.

*Bulinus globosus*, *B. forskalii* and *B. truncatus* were confirmed as transmitting *S. bovis* supporting previous reports that *S. bovis* can utilize a wide variety of *Bulinus* hosts [30, 66]. Conversely, *S. haematobium* and the *S. haematobium* group hybrids appeared more specific and were only transmitted by *B. truncatus* (Table 3). This is consistent with historical findings from the region showing that *B. globosus* and *B. forskalii* were not compatible with *S. haematobium* [34] and questions the previous reports of *B. forskalii* as infected with *S. haematobium*, which could have been morphologically confused with *B. senegalensis* [31, 33]. These findings also include definitive evidence for *B. globosus* acting as a natural intermediate host for *S. bovis* in Niger, since some speculation has surrounded the involvement of this species in transmission of *S. bovis* in West Africa [15, 67–69]. Additionally, although the sample sizes were biased towards *S. bovis*, the *cox*1 data obtained from schistosome cercariae suggest that the mitochondrial *cox*1 diversity is higher for *S. bovis* compared to *S. haematobium,* supporting observations from other studies [29, 50].

Multiple cercarial genotypes were shed from many of the infected *Bulinus* spp., the number of which differing by snail species, with *B. truncatus* shedding the most. However, the sample sizes of *B. forskalii* and *B. globosus* were very small in comparison to *B. truncatus* and so this observation may change with increased sample sizes of these species. Some of the infected snails had multiple schistosome species/strain infections, confirming that they had been infected multiple times by miracidia of different species/strains. However, the majority of infected *Bulinus* spp. shedding multiple cercarial genotypes had single-species infections, suggesting that they had either been infected by multiple miracidia of the same species, or alternatively that different genotypes may have arisen through genetic mutations during clonal parasite replication from a single miracidial infection. Near identical MLG cercariae observed from individual snails has been identified previously for *Schistosoma japonicum* [70–73], with the conclusion that somatic mutation occurs during schistosome sporocystogenesis, resulting in genetically different cercariae originating from a single miracidium. This has also been shown for *S. mansoni*, with significant intraclonal variation of cercariae resulting from single miracidial snail infections [74], and also sporocysts cultured *in vitro*, suggesting that mitotic recombination events occur during intramolluscan larval development [75]. For *S. haematobium*, intramolluscan replication of daughter sporocysts does occur and has been observed in *B. truncatus* [76]; however, the occurrence of mitotic recombination and/or somatic mutation during replication has not been investigated.

If our MLG data do correlate to multiple individual miracidial infections, it is clear that multiple *S. bovis* infections are high in these snails, suggesting that these are high transmission zones, or that *S. bovis* egg deposition is focally more concentrated than that of humans at transmission sites. This is also supported by the finding of all three of the snail species (*B. truncatus*, *B. forskalii* and *B. globosus*) being infected; however, only the former two species harboured high numbers of cercarial genotypes, with *B. globosus* only emitting cercariae of a single genotype (Fig. 2). This may be due to a low sample size of *B. globosus* (*n* = 4), or that this snail species is less suitable for *S. bovis* transmission in this region compared with *B. truncatus* and *B. forskalii* [24, 32, 33, 35]. Comparisons between schistosome species also showed that compared to *S. bovis*, *S. haematobium*/hybrid infections only showed few (≤ 3) cercarial genotypes from individual *B. truncatus* (Fig. 2). Although this again may be a consequence of sample size and the fact that far fewer snails were found shedding *S. haematobium* and/or the hybrids, it might also reflect biological species differences during intramolluscan development, such as variation in sporocystogenesis regulatory mechanisms [77–79].

Interestingly, despite co-endemic foci existing between *S. haematobium*, *S. bovis* and the *S. haematobium* group hybrids in four villages, no snail co-infections were found involving *S. bovis* and *S. haematobium*, or *S. bovis* and the *S. haematobium* group hybrids, the latter being previously reported in Côte d’Ivoire [30]. Co-infections involving *S. haematobium* and *S. haematobium* group hybrids were found however. The lack of *S. haematobium* and *S. bovis* co-infections in the same snails may be due to the highly focal transmission within the villages with transmission sites being used either by humans or cattle, but not both. Sympatric transmission of *S. haematobium* and *S. bovis* was only detected at two out of the 24 transmission sites investigated in our study but no co-infections with these two species were found in the snails (Additional file 1: Table S1).

Alternatively, complex intramolluscan mechanisms may inhibit the occurrence of this co-infection, such as the antagonism/relationship that has been observed between *S. mansoni* and *S. haematobium* with *Calicophoron* spp. trematode parasites [80, 81], or the induced immunoregulation and adaptive immunity of the snail during multiple schistosome infection challenges [82–85], including those of the same *Schistosoma* species. The role that hybridisation plays in relation to these co-infections adds an extra element of complexity, with *S. haematobium* group hybrids being observed to co-infect with both *S. bovis* [30] and *S. haematobium*, signifying the expanded range of compatibility between schistosome hybrids and their intermediate snail hosts warranting further investigation.

Several limitations of the present study are worth considering while making conclusions. First of all, just over a third of infected *Bulinus* spp. and their associated schistosomes collected in the field could not be included due to sample degradation reducing the samples available for this study. The inclusion of these collections in the present study under better circumstances may have revealed other host-parasite relationships not considered here. Secondly, for several of the *Bulinus* spp. included, *cox*1 sequences to distinguish snail species could not be analysed due to the amplification of non-coding DNA or poor sequencing results. Although based on morphological observations of the shell these were attributed to *B. truncatus* and *B. forskalii*, there may be hidden genetic diversity within these species currently undetermined. In addition, the two snails morphologically identified as *B. forskalii* may be those of a closely related species such as *B. senegalensis* reported as present in Niger [34]. Thirdly, it should be reiterated that the inferences made in the present study regarding multiple schistosome miracidia infections per *Bulinus* spp. were established primarily using microsatellite markers alone and are therefore in need of further support. As aforementioned, we need to conduct controlled experimental infections of *Bulinus* spp. with *Schistosoma* spp. to establish how these microsatellite loci may be affected during intramolluscan replication when somatic mutations may occur. During such infection experiments, microsatellite markers should be analysed alongside other variable regions of schistosome DNA (as was demonstrated for one snail in the present study where different *cox*1 sequences haplotypes from each MLG confirmed multiple infections) to give further support for the occurrence of multiple miracidia infections and/or somatic mutation in field collected intermediate hosts.

## Conclusions

Detailed snail and schistosome sampling coupled with molecular analyses has advanced our understanding of human and bovid schistosomiasis transmission in the Niger River Valley region. Schistosomes found to infect humans (*S. haematobium* and *S. haematobium* hybrids) were much less abundant than those causing veterinary schistosomiasis (*S. bovis*) across the region. No genetic overlap was observed between human and bovine schistosomes, supporting population structure and division between *S. haematobium* and *S. bovis*. *Bulinus truncatus*, the most abundant snail species [38], was involved in transmission of all schistosomes, whilst the less abundant *B. forskalii* and *B. globosus* were only involved in the transmission of *S. bovis.* Our data suggest that species-specific biological traits may exist in relation to co-infections, snail-schistosome compatibility and intramolluscan schistosome development which might affect transmission dynamics and genetic outcomes of the different schistosome populations. Further experimental studies investigating the genetic outcomes of schistosome intramolluscan replication are recommended to shed light on this relatively unknown part of schistosome biology. Even in highly endemic settings such as the Niger River Valley, the scarcity of human-infecting schistosomes to the comparatively abundant livestock schistosomes, shows the necessity from a public health viewpoint to identify species accurately to assess the presence and level of human schistosomiasis transmission. If species identifications of snails and their associated schistosomes can be sustained in future control programmes, interventions to control *S. haematobium* in this Niger River Valley region can be tailored to areas where urogenital schistosomiasis transmission is taking place. This will enable resources such as implementing targeted snail control, now listed as a critical action by the World Health Organization for combatting schistosomiasis [19], to be focussed on high-risk areas for urogenital schistosomiasis and not those contributing to the maintenance of bovine schistosomiasis.

## Supplementary information

**Additional file 1: Table S1.** Complete dataset used in this study providing snail collection information (from malacological survey), *Bulinus* and *Schistosoma* species identifications (including SNPs where necessary) and *Schistosoma* microsatellite loci data including genotypes identified from each snail.

**Additional file 2: Table S2.** PCR primers and cycling conditions for amplification of mitochondrial and nuclear DNA regions of *Schistosoma* spp. and Folmer *cox*1 region of *Bulinus* spp.

**Additional file 3: Table S3.** Microsatellite loci and primers used for identification of *Schistosoma* cercariae genotypes.

**Additional file 4: Table S4.** Expected and observed *Schistosoma* species-specific SNP positions in nuclear genes sequenced from the cercariae studied.

**Additional file 5: Figure S1.** Haplotype network of unique *cox*1 haplotypes found for each *Bulinus* species. Generated using PopART [40].

**Additional file 6: Figure S2.** Bayesian analysis of the partial mitochondrial *cox*1 haplotype dataset from *Bulinus* spp. The branch length scale bar indicates the number of substitutions per site.

**Additional file 7: Figure S3.** Pearson’s correlation test comparing number of cercarial genotypes to the cercarial microsatellite profiles amplified/analysed per *Bulinus* spp. Pearson’s correlation coefficient, *r* = 0.04.

## Data Availability

All of the data generated and/or analysed during this study are included in the article and its additional files. The sequence data generated and/or analysed during the present study are available on GenBank under the accession numbers MT272322-MT272334 and MT272336-MT272340.

## Abbreviations

MLG: multilocus genotype
SCORE: Schistosomiasis Consortium for Operational Research and Evaluation
PCA: principal components analysis
*S.h*-*b*: *S. haematobium*-*bovis* hybrid
*S.h*-*b*-*c*: *S. haematobium*-*bovis*-*curassoni* hybrid
