# Supplementary figures and images for: Interactions between Schistosoma haematobium group species and their Bulinus spp. intermediate hosts along the Niger River Valley
Source: Parasit Vectors. 2020 May 24;13:268. doi: 10.1186/s13071-020-04136-9 (PMC7247258; doi:10.1186/s13071-020-04136-9)

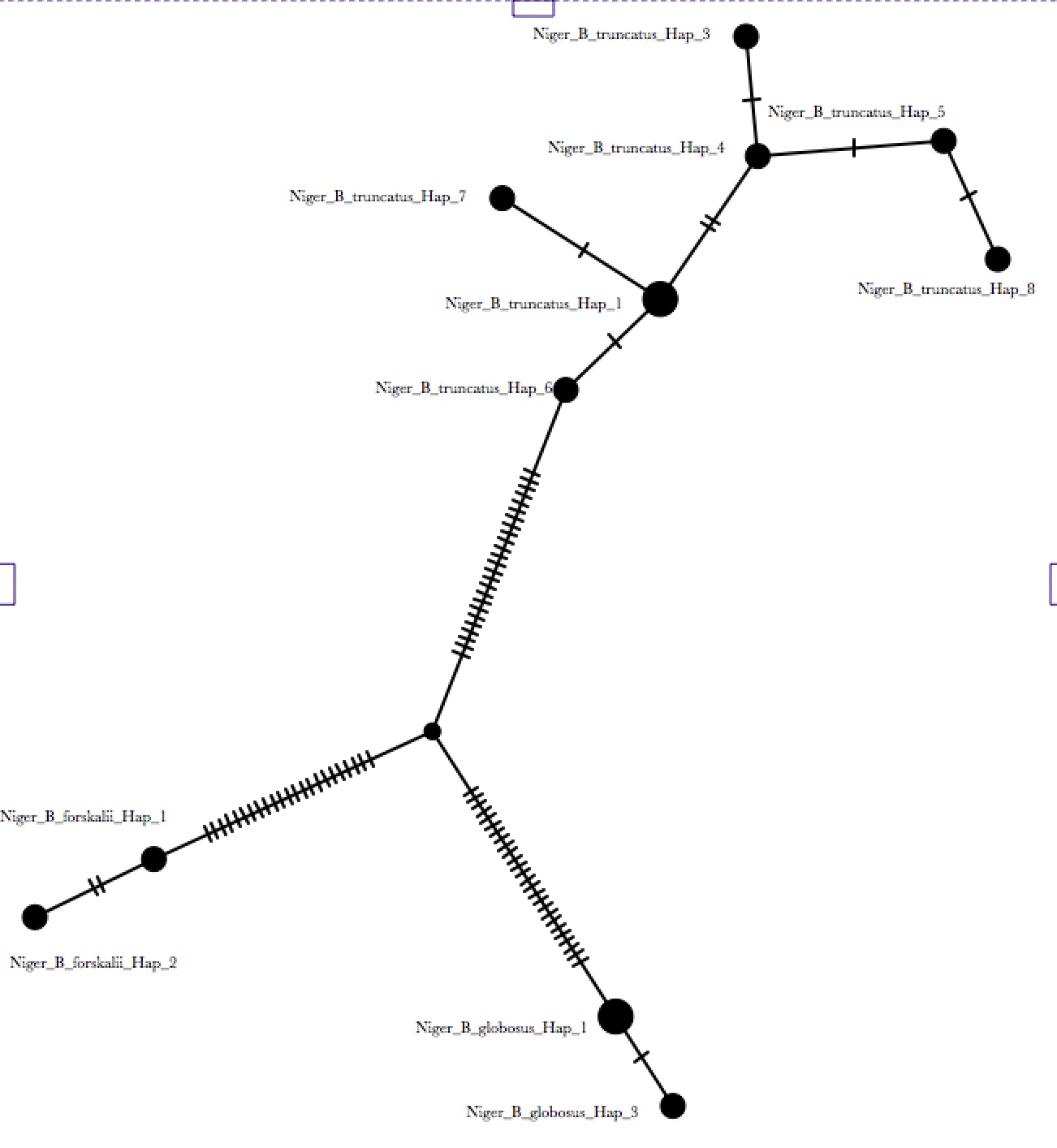

Supplement: Supplementary file 5 — Additional file 5: Figure S1. Haplotype network of unique cox1 haplotypes found for each Bulinus species. Generated using PopART [40]. [file 13071_2020_4136_MOESM5_ESM.jpeg]

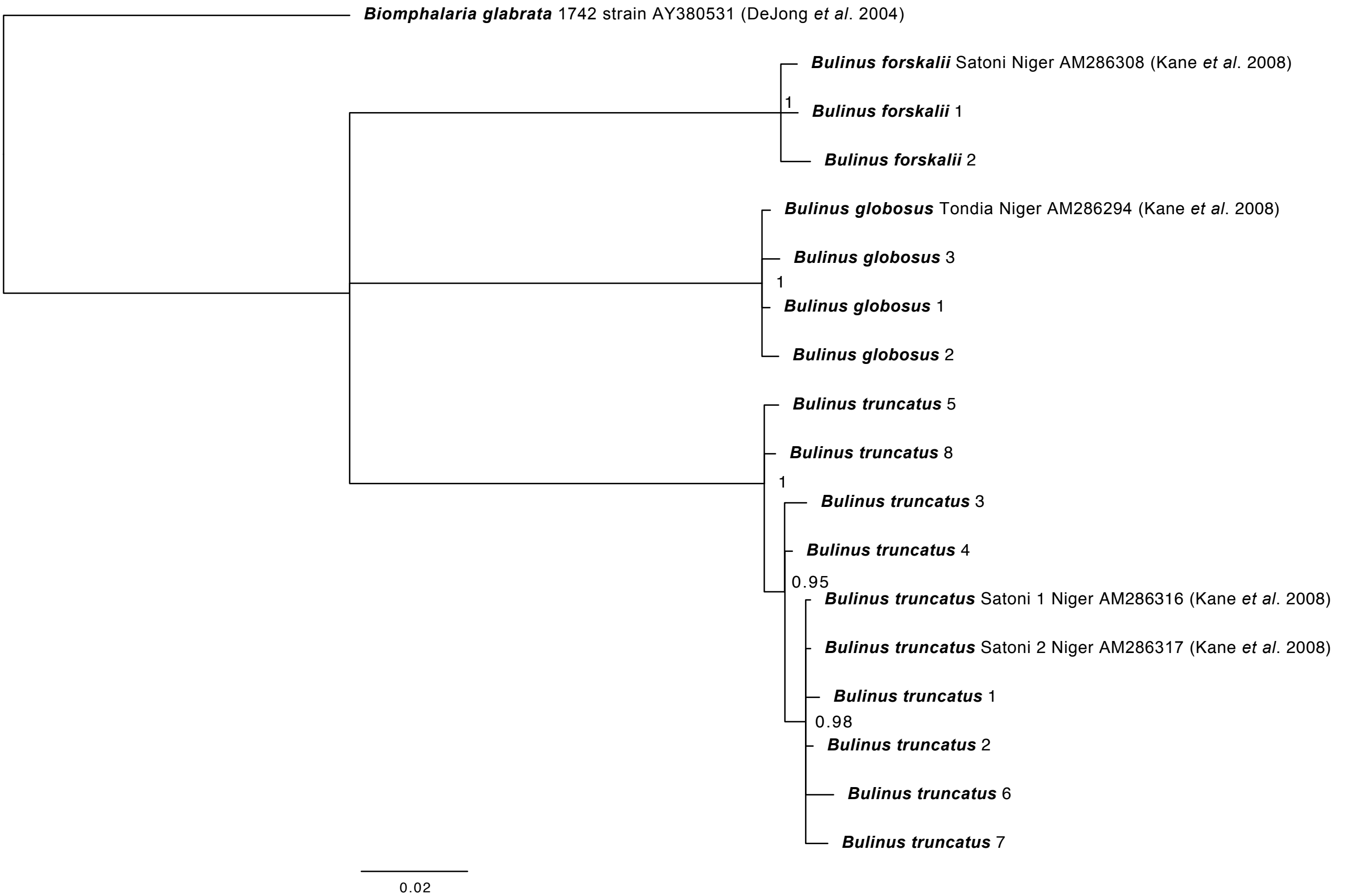

Supplement: Supplementary file 6 — Additional file 6: Figure S2. Bayesian analysis of the partial mitochondrial cox1 haplotype dataset from Bulinus spp. The branch length scale bar indicates the number of substitutions per site. [file 13071_2020_4136_MOESM6_ESM.pdf]

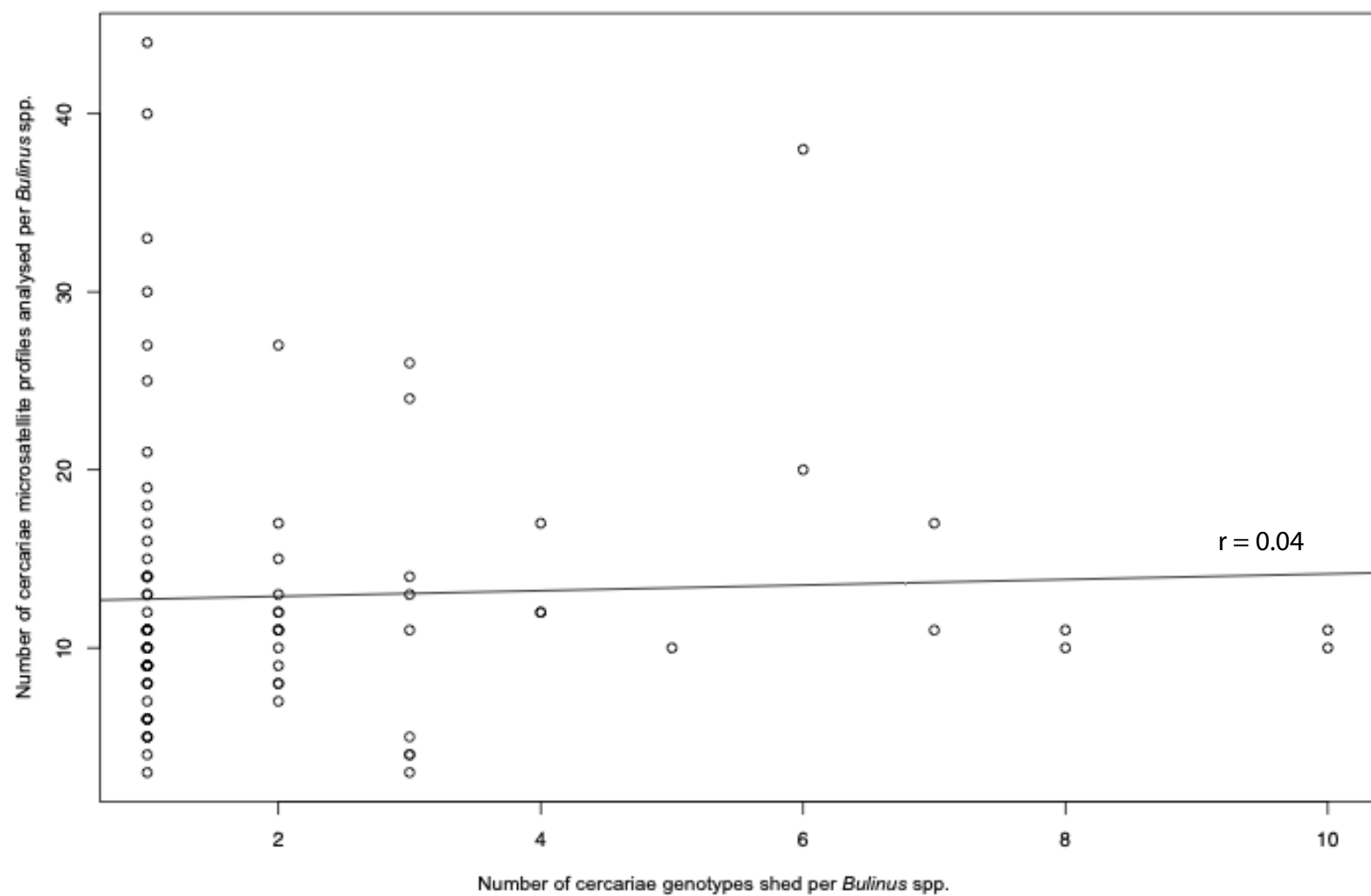

Supplement: Supplementary file 7 — Additional file 7: Figure S3. Pearson’s correlation test comparing number of cercarial genotypes to the cercarial microsatellite profiles amplified/analysed per Bulinus spp. Pearson’s correlation coefficient, r = 0.04. [file 13071_2020_4136_MOESM7_ESM.pdf]
